# Revisiting the Radiobiology of Targeted Alpha Therapy

**DOI:** 10.3389/fmed.2021.692436

**Published:** 2021-07-27

**Authors:** Jean-Pierre Pouget, Julie Constanzo

**Affiliations:** Institut de Recherche en Cancérologie de Montpellier (IRCM), Inserm U1194, Université de Montpellier, Institut Régional du Cancer de Montpellier (ICM), Montpellier, France

**Keywords:** radiobiology, bystander, non-targeted effects, lipid rafts, cGAS-STING, targeted alpha radiotherapy, targeted alpha particle therapy

## Abstract

Targeted alpha therapy (TAT) using alpha particle-emitting radionuclides is in the spotlight after the approval of ^223^RaCl_2_ for patients with metastatic castration-resistant prostate cancer and the development of several alpha emitter-based radiopharmaceuticals. It is acknowledged that alpha particles are highly cytotoxic because they produce complex DNA lesions. Hence, the nucleus is considered their critical target, and many studies did not report any effect in other subcellular compartments. Moreover, their physical features, including their range in tissues (<100 μm) and their linear energy transfer (50–230 keV/μm), are well-characterized. Theoretically, TAT is indicated for very small-volume, disseminated tumors (e.g., micrometastases, circulating tumor cells). Moreover, due to their high cytotoxicity, alpha particles should be preferred to beta particles and X-rays to overcome radiation resistance. However, clinical studies showed that TAT might be efficient also in quite large tumors, and biological effects have been observed also away from irradiated cells. These distant effects are called bystander effects when occurring at short distance (<1 mm), and systemic effects when occurring at much longer distance. Systemic effects implicate the immune system. These findings showed that cells can die without receiving any radiation dose, and that a more complex and integrated view of radiobiology is required. This includes the notion that the direct, bystander and systemic responses cannot be dissociated because DNA damage is intimately linked to bystander effects and immune response. Here, we provide a brief overview of the paradigms that need to be revisited.

## Introduction

Targeted alpha therapy (TAT), based on alpha particle-emitting radionuclides, has become popular in the last decades after the approval of Xofigo (^223^RaCl_2_) and the encouraging results obtained for several radiopharmaceuticals under investigation. However, the biological advantages of alpha particles compared with gamma/X rays have been known for more than 60 years. Superficially, the radiobiology of alpha particles is well-understood. Because of their double-positive charge (${}_{2}^{4}$He^2+^), alpha particles deliver dense ionizations along a linear track, also known as linear energy transfer (LET), ranging from 50 keV/μm to a maximum of 230 keV/μm at the Bragg Peak; Since their energy is between 4 and 10 MeV, their ranges do not exceed 100 μm in water-equivalent tissues and 40 μm in bone. Therefore, alpha particles might offer lower dose conformity compared with beta particles or photon beams, but with less irradiation of normal tissues. It is also generally admitted that the nucleus is the sensitive target and that complex and thus unrepairable DNA double strand breaks (DSB) explain the high cytotoxicity of these particles. Consequently, decreasing the dose rate and fractionating the dose, which promote DNA repair, have no effect on the alpha particle killing potential. Moreover, the presence of O_2_, which plays a central role in reactive oxygen species (ROS) production during water radiolysis through the production of peroxy radicals, is not required for alpha particle mode of action that mostly involves direct ionization. Therefore, alpha particles are suitable for treating hypoxic tumors. In addition, as their range in tissue is short, they should be dedicated to the treatment of single cancer cells and micrometastases.

However, the most striking results with TAT have been observed in much larger tumors. Kratochwil et al. reported the first-in-human trial showing the efficacy of ^213^Bi-DOTATOC TAT in patients with progressive advanced neuroendocrine tumor liver metastases, pretreated with beta emitters (1). Two years later, the same group observed in patients with metastatic castration-resistant prostate cancer, the decrease of the tumor burden in liver and of disseminated bone marrow metastases after TAT using a prostate-specific membrane antigen (PSMA) ligand labeled with ^225^Ac (2). These tumors could be monitored using PET/CT, suggesting that TAT was effective in quite large tumors (e.g., >1 cm) and not only in single cells and micrometastases. Another received idea, also invalidated by this study, is that the physical half-life of the radionuclide (^225^Ac) must match the biological life of the vector (PSMA ligand).

## The Target Cell Paradigm

The most striking observation when comparing the cytotoxicity of alpha particles, gamma/X rays and beta particles is that for the same radiation dose, alpha particles are much more deleterious. This means that they have a higher relative biological effectiveness (RBE). This was first described by Zirkle when he irradiated fern spores with alpha particles (3). He also identified the cell nucleus as the major target of radiation lethality and demonstrated the importance of LET. From the late 1950s, radiobiology development has been associated with the clonogenic assay, described by Puck and Marcus (4), to measure reproductive death in irradiated cells. The clonogenic assay allows determining the capacity of irradiated cells to divide and to form a macroscopic colony in culture. Clonogenic survival is defined as the ratio between the number of cells that form colonies (n) and the number of seeded cells (n0). The number of colonies must be corrected relative to the number of colonies measured in non-irradiated samples. For their first experiments using alpha particles, Barendsen and Beusker developed an irradiation system with a ^210^Po (E_alpha_ = 5.3 MeV) source that corresponded to an alpha range of about 37 μm in water, and in which extra-thin material (Melinex film) was placed between the source and the cells (5–7). By using a dosimetric approach, they showed that the clonogenic survival of cells exposed to high LET (alpha) particles follows an exponential law described by:

(1) $$\begin{array}{r} \text{n}/\text{n}0={\text{e}}^{-SD} \end{array}$$

Conversely, they obtained a “less simple shape” for X- and beta radiation. *D* is the number of particles/μm^2^ and *S* is a factor of proportionality in μm^2^. The RBE was about 2.5 with high doses and about 6 with lower radiation doses. From the survival curve equation, it was possible to determine that *S* corresponds to 42 μm^2^. As the irradiation flux is perpendicular to the bottom of Petri dishes, this means that cells contain a sensitive area of 42 μm^2^, which matches the nucleus cross-section. This ballistic view of alpha particle killing effect was further developed in mathematical models that considered the random and physical nature of the interactions between radiation and biological matter. Cell death was directly correlated with particles traversing the nucleus. This theory was called “one hit one target,” but the relationship was then slightly modified to consider the vital region in the nucleus. Thus, cell death was considered to be related to the probability of hitting this vital target (αD). A Poisson distribution was used to express the probability density function that describes the number of hits to vital cellular targets. The probability for a cell to have k lethal hits could be expressed as follows:

(2) $$\begin{array}{r} \text{Probability }\left(\text{X}=\text{k hits}\right)=\frac{\alpha D^{k}}{k!}\;e^{-\alpha D} \end{array}$$

The probability for a cell to survive would be to have 0 lethal hit:

(3) $$\begin{array}{r} \text{Probability }\left(\text{X}=0\text{ hits}\right)=\frac{\alpha D^{0}}{0!}\;e^{-\alpha D\;}=e^{-\alpha D} \end{array}$$

The value *D*_0_ is the dose (Gy) leading to the average number of one lethal hit per cell (αD = 1). A dose *D*_0_ reduces cell survival from 1 to 0.37 (i.e., to e^−1^).

Explaining the effect of low LET radiation was slightly more complex and the linear quadratic model was proposed:

(4) $$\begin{array}{r} \text{SF}={\text{e}}^{-\alpha D-\beta D^{2}}\text{ or ln}\left(\text{SF}\right)=-\text{α}D-\beta D^{2} \end{array}$$

where α represents the cell intrinsic radiosensitivity (1 hit = 1 lethal event) and β the cell sparing capacity (i.e., repair) of the cells.

## From Physical and Chemical Events to DNA Damage

Alpha particle cytotoxicity, measured with the clonogenic assay, is explained by how particles interact with biological matter. Ionizing radiation may release their energy through two pathways. The first one (called direct effect) consists of direct energy transfer to biomolecules (DNA, lipids, proteins), leading to their ionization, namely the loss of one electron and the formation of radical cations. In DNA, guanine has the lowest oxidation potential. Then, even if a radical cation is produced on another base or sugar moiety, a fast electron transfer reaction occurs from guanine to the generated radical cation, repairing the initially produced radical and generating a guanine radical cation (G^°+^). This unstable cations can give rise to two guanine chemical modifications: 8-oxo-7′8-dihydro-2′-deoxyguanosine (8-oxodGuo) following oxidation and the corresponding formamidopyrimidine derivative FapydGuo. Therefore, oxidation of 2′-deoxyguanosine is considered the hallmark of direct DNA ionization.

In the second pathway (called indirect effect of ionizing radiation), energy is transferred to water, the most ubiquitous molecule, that is then dissociated into ROS species among which the hydroxyl radical HO° is the most reactive. HO° reacts with the biomolecules present in the cell. In the case of DNA, single strand breaks, DSBs, base damage, abasic site, and DNA-protein crosslinks can be produced, at predefined yields, upon low LET radiation. The contribution of the direct and indirect effects depends on the particle LET.

## The DNA Centered Approach: Alpha Particles Generate Multiple Damage Sites in DNA

Because of their high LET, alpha particles produce locally high density of ionization in biological matter. Therefore, water radiolysis leads to the production of high concentrations of radicals, including HO°, that will tend to recombine before attacking biomolecules. Direct effects should be predominant when using high LET particles, such as alpha particles. Indeed, we showed that the yield of some base damage (involving mostly HO°) is lower with high LET radiation than with γ-rays, likely because of radical recombination (8). However, we found that 8-oxodGuo, the signature of a direct effect, is not the most frequent lesion with high LET radiation. This indicates that the contribution of indirect effects to the high LET particle-induced damage could be larger than what thought (8), but this hypothesis needs to be further investigated. Another feature of high LET radiation is that multiple direct ionization events on DNA are accompanied by the production of damage clusters (i.e., multiple damage sites). These are defined as two or more modifications per helix turn (9, 10), and DNA DSBs are one of the best examples. It has been shown that alpha particles activate ATM. This is a master kinase in the DNA damage response and is involved in many cell functions that are induced in response to irradiation, including cell cycle arrest, apoptosis and DNA repair (11, 12). DNA repair is mediated by the non-homologous end-joining (NHEJ) system, which is active in all cell cycle phases, and by homologous recombination (HR) in the S/G2 phase when cells have duplicated their DNA. However, cells cannot repair most of the complex lesions, and misrepaired lesions could lead to genomic instability or cancer (13). Consequently, alpha particles are highly deleterious and this also explains their high RBE values.

The DNA centered view of radiobiology was further comforted in the 1980–1990s by the finding that the level of unrepaired DNA lesions can be correlated with the cell sensitivity to radiation. However, events occurring in the cytoplasm or at the cell membrane also have consequences on nuclear DNA. As it is not possible to discriminate between nuclear damage caused by nuclear and non-nuclear events, assessing nuclear damage could overestimate the contribution of nuclear hits to the final cell outcome.

## Revisiting Paradigms: Subcellular Targets

Besides the DNA centered approach, in the last two decades, many studies have promoted a more integrated view of TAT radiobiology. They propose that other subcellular targets, including the cell membrane, mitochondria, and lysosomes (14), participate in the response to radiation. It should not be forgotten that alpha particles must traverse the cell membrane, cytoplasm including organelles, and nuclear membrane to reach the nucleus. Therefore, they might have some effects in these compartments, and the contribution of these extranuclear effects to cell death needs to be accurately assessed.

The study of extranuclear targets has been facilitated by the development of microbeam technologies. The first microbeam device was used by Zirkle and Bloom, and consisted of a 2 MV Van de Graaff accelerator that delivered protons (15). Today, new-generation microbeam devices allow reducing the radiation beam to sub-cellular dimensions using collimation assemblies and electromagnetic focusing (16). External alpha particle microbeam irradiation has been and is a very attractive tool for exploring the radiobiology of alpha particles at the subcellular scale (17), although different from TAT in terms of the used dose and dose rate, and the absence of vector (18, 19). The first reports using these microbeams in the 1990–2000s indicated that direct DNA damage hits and the whole cell should be considered as a sensor of radiation exposure (20, 21). Interestingly, dosimetric approaches confirmed the role of extranuclear targets during alpha particle irradiation (22). Microbeam technology has been also very useful to investigate bystander effects measured in neighboring non-irradiated cells (see below).

## Alpha Irradiation of the Cell Membrane

Biological membranes are ubiquitous in cells and organelles. However, only few studies have investigated the cell membrane response to irradiation [reviewed in (23–25)]. The cell membrane is a 10-nm thick, orientated and dynamic bilayer constituted of lipids (30–80%; glycerophospholipids, sphingolipids), proteins (20–60%), and carbohydrates (0–10%). As molecules can move in the plane of the membrane (26), any fluidity change may have biological consequences. Lipids contain polyunsaturated fatty acids, and therefore they can be oxidized by HO° to generate lipid hydroperoxides (27) that are then degraded to reactive aldehyde products, including malondialdehyde and hydroxyalkenals (e.g., 4-HNE), with great reactivity toward DNA, proteins, and lipids. Lipid peroxidation disrupts the cell membrane conformation and biological functions.

**Figure 1:**
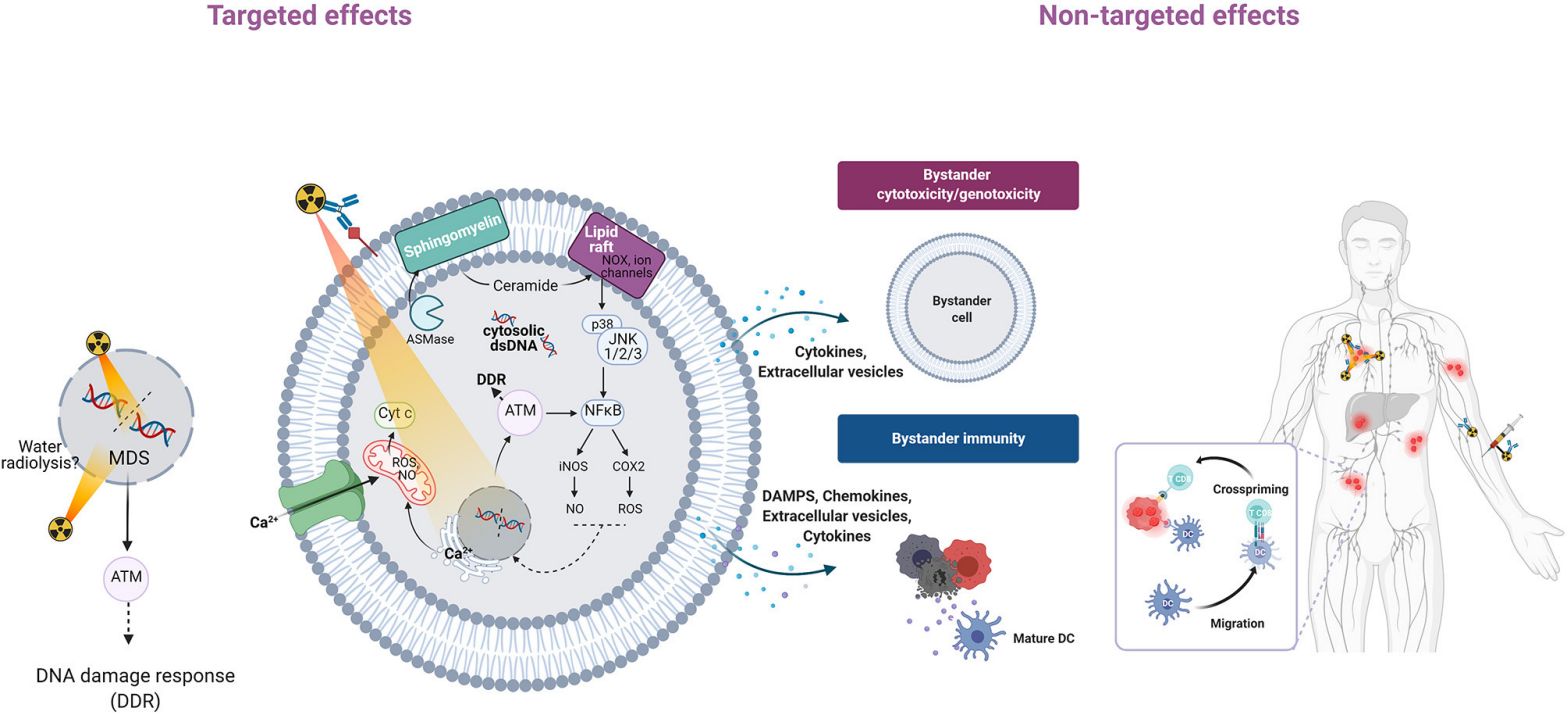
Overview of targeted and non-targeted effects of radiation. Alpha-particle irradiation of a cell population can induce targeted effects (in cells hit directly by particles) and non-targeted effects (in non-irradiated cells). Non-targeted effects can be detected at short distance (bystander effects) or at long distance (systemic effects). Genomic instability, another class of non-targeted effects, is not described here. In irradiated cells (targeted effects), alpha particles induce DSBs and non-DSB clustered DNA lesions (MDS) that are detected by ATM and activate the DNA damage response (11, 12). Alpha-particle irradiation of the cell membrane generates lipid peroxidation products (4-HNE, 4-hydroxy-2-nonenal; MDA, malondialdehyde) from polyunsaturated fatty acids (PUFA) (43). Alpha-particle irradiation can also activate acid sphingomyelinase (ASMase) and this leads to the rapid formation of ceramide through hydrolysis of sphingomyelin, a cell membrane phospholipid (38, 42). Ceramide-enriched large domains (lipid rafts) are formed by aggregation of ceramide, leading to activation of the mitogen-activated protein kinase (MAPK) pathway and its downstream effector, nuclear factor kappa B (NF-κB) (41). NF-κB induces the transcription of target genes, such as those encoding cytokines, COX-2, and inducible nitric oxide synthase (iNOS), followed by production of ROS and nitric oxide (NO) that contribute to oxidative stress (44). Irradiation can also increase the intracellular Ca^2+^ level (45) through release from the endoplasmic reticulum via calcium release mechanisms (46). Ca^2+^ can in turn activate protein kinase C, the MAPK pathway and transcription factors (NF-κB, AP1) that promote various downstream pathways (iNOS, COX-2). Mitochondria also are affected by alpha-particle irradiation (47–52). Ca^2+^ can be taken up by mitochondria, leading to ROS and RNS increase, mitochondrial DNA damage, altered ATP synthesis, mitochondrial depolarization, and release of cytochrome C and caspase 3. Mitochondrial fission also has been observed. Targeted cells can communicate with bystander cells through gap junctions or through the release of soluble factors. Extracellular vesicles, including exosomes, containing nucleic acids, lipids and proteins, might be released, and contribute to bystander immunity. Systemic effects may involve the immune system through the release of DAMPs that are recognized by antigen-presenting cells (e.g., dendritic cells, DC, that present antigenic peptides to CD4^+^ and CD8^+^ T lymphocytes for immune response activation). Altogether these effects contribute to tumor cell death.

In reality, cell membranes are not just a fluid mosaic explained by the low melting temperatures of phospholipids existing in a liquid disordered phase (26). Indeed, domains of about 50 nm in size that contain sphingolipids and are resistant to detergents have been identified in cell membranes. Their origin is explained by the higher melting temperature of sphingolipids (e.g., ceramide) and their tendency to interact with each other via hydrophilic interactions between lipid head groups. These domains, called lipid rafts, are stabilized by cholesterol (28, 29). Moreover, they can be extended into large domains by the addition of ceramide. This process might be favored by radiation because it can activate acid sphingomyelinase that catalyzes sphingomyelin hydrolysis into ceramide (30, 31). Then, ceramide aggregates into ceramide-enriched large platforms (lipid rafts) that contain ions channels, NADPH oxidase, receptors and enzymes, but it can also be a second messenger of apoptosis (32, 33). Ceramide is generated in the outer leaflet of the cell membrane, but can flip into the cytosolic side where it activates cytosolic phospholipase A2, protein phosphatase 2 and protein phosphatase 1. These serine/threonine phosphatases in turn activate MAP kinases, including extracellular signal-related kinase (ERK) 1 and 2, ERK5, c-Jun N-terminal kinase (JNK) 1 and 2, p38, protein kinase C isoforms, retinoblastoma proteins, and BCL-2 (34). Finally ceramide-enriched large domains participate in many cellular signaling pathways implicated in the regulation of potassium (35) and calcium (36) channels, cell death, cell survival, and inflammatory response. Several studies have reported the cell membrane role in alpha particle irradiation-induced cell death. For instance, Narayanan et al. suggested that plasma membrane-bound NADPH-oxidase is mainly responsible for the increased intracellular ROS production and that ROS response does not require direct nuclear or cellular hits (37). Nagasawa et al. showed by incubating CHO cells with filipin, a drug that disrupts lipid rafts and effectively inhibits membrane signaling, that signals arising in the cell membrane are involved in the bystander effects of low-fluence alpha particles (21). Similar findings were reported by Hanot et al. in osteoblastic cells exposed to alpha particle irradiation using a microbeam device (38). Seideman et al. showed that alpha particles produced by ^225^Ac-labeled antibodies can activate the sphingomyelin pathway to induce apoptosis (39). We demonstrated, using radiolabeled antibodies, that the cell membrane is a sensitive target of Auger (^125^I) (40, 41) and alpha (^213^Bi, ^212^Pb/^212^Bi) particle irradiation [(42); Figure 1]. We also showed that lipid raft formation and downstream signaling pathways participate in the cytotoxic and genotoxic effects in irradiated and also bystander cells. For example, although ^125^I is localized at and mostly irradiates the cell membrane, we found DNA damage also in the nucleus. When using Auger and alpha particle emitters, lipid raft formation contributes to nuclear DNA damage through signaling pathways that involve AKT, ERK1/2, p38 kinase, and JNK, together with phospholipase C-c, proline-rich tyrosine kinase 2, and paxillin (involved in Ca^2+^ fluxes) (40). Finally, we demonstrated that radiation-induced membrane modifications lead to cell death, and that inhibition of lipid raft formation restores cell survival.

## Alpha Irradiation of Cytoplasm and Mitochondria

The first studies on cytoplasm irradiation were done by Zirkle and Bloom in 1953 (15), and in 1970, Munro showed that the cytoplasm is much less sensitive to irradiation than the nucleus (53). The notion that cytoplasm is not sensitive to radiation has been progressively reconsidered, and cytoplasm irradiation has been associated with bystander effects in studies using microbeams. Several groups (20, 43, 47, 48) found that targeted cytoplasmic irradiation induces oxidative DNA damage and also lipid peroxidation, as shown by the increased formation of 4-hydroxynonenal (43).

The cytoplasm contains many different organelles among which mitochondria represent up to 25% of the cell volume and constitute a prominent radiation target. The number and biogenesis of mitochondria are modified by alpha-particle irradiation through upregulation of genes encoding mitochondrial biomarkers (LONP1, TFAM) and mitochondrial DNA-encoded genes (*MT-CYB, MT-RNR1*) (45). Mitochondria are polarized organelles with a membrane potential (negative inside) that plays a crucial role in energy homeostasis. Loss of this potential is accompanied by cytochrome C release and then caspase activation involved in apoptosis. Moreover, mitochondria contain a circular double-stranded genome (mitochondrial DNA) that encodes proteins, as well as transfer and ribosomal RNAs. Mitochondrial DNA can be damaged by alpha particles and this affects mitochondrial functions (54, 55). It was also shown that high LET irradiation with carbon ions leads to mitochondria depolarization (49), and alpha-particle microbeam irradiation causes their fragmentation through mitochondrial fission that requires the mitochondrial fission protein dynamin-related protein 1 (DRP1) (50). Mitochondrial fission also participates in the phosphorylation of AMP activated protein kinase (AMPK) and in the activation of ERK1/2 signaling pathways (48). In addition, DRP-1 has a role in autophagy for the degradation of dysfunctional mitochondria to maintain the cellular energy homeostasis. Conversely, mitochondrial impairment following irradiation contributes to the persistence of oxidative stress through dysfunction of respiratory complex I, leading to intracellular increase in ROS production and mitochondrial DNA damage.

Mitochondrial damage plays a role also in bystander effects (see below). Indeed, signals detected in irradiated mitochondria could be transmitted to neighboring mitochondria via a reversible Ca^2+^- dependent mitochondrial permeability transition that results in enhanced ROS/reactive nitrogen species (RNS) generation (56). On the other side, impaired mitochondrial function in alpha-particle irradiated cells is associated with reduction in DNA mutations in bystander cells (51). Moreover, functional mitochondria are required for 53BP1 focus formation in directly hit and in bystander cells during cytoplasmic irradiation, independently of the dose to the nucleus (47).

## Revisiting the Target Cell Theory: Involvement of Bystander Effects

Another change in the last 20 years concerned the reanalysis of the target cell theory (i.e., only cells traversed by particles can be killed) after the description of non-targeted effects. *In vitro*, non-targeted effects commonly comprise bystander effects, genomic instability, adaptive response, and low-dose hypersensitivity. *In vivo*, they also include long-range effects induced by the immune response activation (i.e., systemic or *abscopal* response to radiotherapy).

Bystander effects are characterized by cytotoxic and genotoxic modifications (DNA damage, chromosomal aberrations) in cells that are located in the proximity of irradiated cells, but that are not traversed by particles. In 1992, Nagasawa and Little were the first to show the involvement of bystander effects in alpha particle irradiation in CHO cells irradiated with low fluences of alpha particles produced by ^238^Pu (57). Their findings were confirmed and expanded by many other researchers (58–61). They observed that sister chromatid exchanges were increased in 30% of cells, although only 1% of nuclei were traversed by particles. This demonstrated that cells do not need to be traversed by particles (dose equal to zero) to be killed and that intercellular communications play a role (59, 60) because bystander effects are inhibited by drugs that block gap junctions, such as lindane (61). In turn, bystander cells also can communicate with irradiated cells (62).

As the cell membrane plays a central role in intercellular communications, many studies focused on its function in bystander effects. For example, in a model in which alpha-particle irradiated human macrophage-like cells (U937 line) are co-cultured with HL-7702 hepatocytes (bystander cells), inhibition of the cell membrane signaling pathway (cAMP transmission) by filipin prevents the protective effect (i.e., reduction of micronuclei) of bystander cells on irradiated cells (62). Hu et al. also showed that incubation of alpha-particle irradiated (^241^Am) fibroblasts with lindane, a drug that blocks gap junctions, strongly reduces the percentage of bystander cells harboring DNA DSBs, suggesting that genotoxic agents are transmitted via gap junctions (62).

We made similar observations by exposing different tumor cell lines (colorectal HCT116, squamous vulvar A-431, ovarian SK-OV-3 cells) to antibodies radiolabeled with an alpha particle emitter. First, we showed that clonogenic survival of cells incubated with conditioned medium from irradiated cells was significantly decreased, and determined that about 30–35% of cells were killed by bystander effects. We also highlighted the role of cell membrane in these bystander effects because irradiation in the presence of filipin or methyl-beta-cyclodextrin (MBCD), two compounds that disrupt lipid rafts, abolished these effects (40, 42). We then confirmed this finding *in vivo* in mice where combining Auger or alpha particle-based targeted radiotherapy with MBCD or pravastatin (inhibitor of cholesterol synthesis) decreased the therapy effect (tumor growth delay) (40, 42). This was accompanied by a decrease in the global DNA damage yield in tumors, indicating that lipid raft disruption has an effect on DNA damage. Moreover, in the tumors collected from these mice, DNA DSBs could be observed up to 1 mm from sites of radioactivity decay (40, 42). Finally, the consequence of these bystander effects *in vivo* is that tumor growth in mice treated with ^212^Pb-labeled monoclonal antibodies was less important than what one might have expected based on voxel dosimetry (42). These results are quite similar to those obtained by Belyakov et al. who using alpha-particle microbeam irradiation of reconstructed skin in a three-dimensional system found that radiation-induced biological effects can be measured also in non-irradiated tissue up to 1 mm from the directly irradiated area (63).

**Figure 2:**
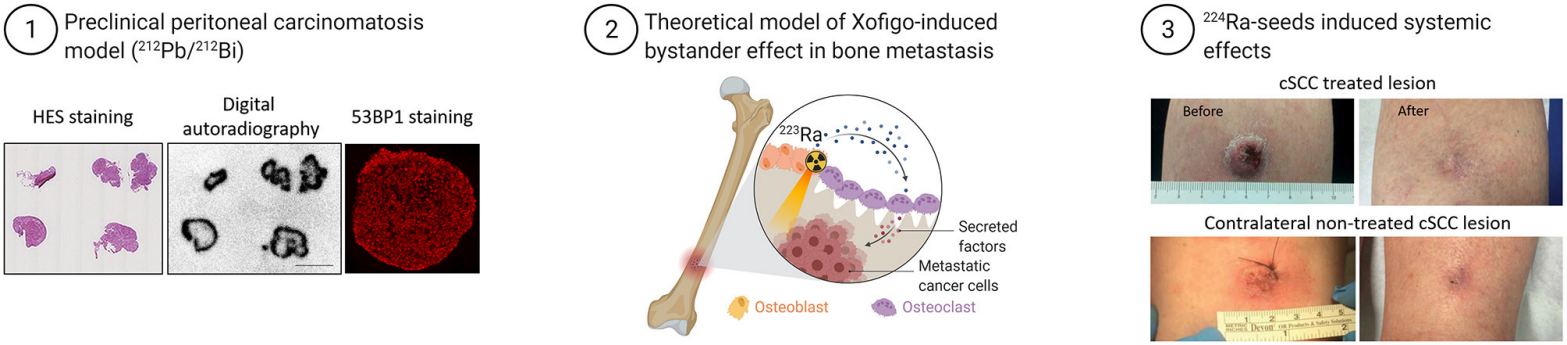
Example of bystander effects at tissue scale and abscopal effects in patients exposed to TAT. Examples of bystander effects: (1) Strong heterogeneous distribution of radioactivity (^212^Pb-labeled monoclonal antibodies) in tumors of mice treated with TAT was unexpectedly accompanied by homogenous distribution of DNA damage, measured by immunofluorescence analysis of 53BP1 expression (*modified from Ladjohounlou et al*.). (2) Model of ^223^RaCl^2^ (Xofigo)-induced bystander effects in a bone metastasis. (3) Systemic effect induced in a patient with cutaneous squamous cell carcinoma (cSCC) who received alpha brachytherapy to one tumor lesion. Surprisingly, another tumor lesion far from the radiation area also was cured (66) (*modified from Bellia et al*.).

Another example of bystander effects comes from studies on ^223^RaCl_2_ that has been approved for patients with metastatic castration-resistant prostate cancer or with symptomatic bone metastases after at least two prior lines of systemic therapy (64, 65). Due to the range of bone sizes (from few millimeters to centimeters in thickness), cancer cells within bones do not receive a uniform dose and may also not be irradiated (Figure 2). In these non-irradiated or only sparsely irradiated cells, the impact of non-targeted effects may be crucial to achieve tumor control and regression. Suominen et al. monitored the localization of alpha particles by autoradiography in mice harboring LuCaP 58 (prostate cancer) cells in the tibia after a single intravenous administration of ^223^Ra (67). They detected ^223^Ra deposits mostly within the bone matrix and especially in the vicinity of osteoblasts, and less frequently co-localized with prostate cancer cells (67). Abou et al. used a mouse model of prostate cancer bone metastases in which osteoblastic (LNCaP) and osteolytic (PC3) cells are inoculated in the tibia to monitor the acute micro-distribution of ^223^Ra by autoradiography (68). They found that ^223^Ra does not localize directly in the tumor cells, but accumulates at the bone surface surrounding the lesion and at active bone modeling/remodeling sites (68). These radiologic features make conventional dosimetry less relevant. Indeed, stochastic variations in the energy deposited in cell nuclei are important because of the microscopic target size, low number of crossing alpha particles, and LET variation along the alpha particle track (69). These results are supported by a recent study indicating the participation of ^223^Ra-induced antiproliferative/cytotoxic bystander effects in delaying the growth of tumor cell xenografts (70). Overall, these preclinical results suggest that the better survival observed in patients treated with ^223^Ra could be explained also by cancer cell death induced by non-targeted effects arising from irradiated osteoblasts/osteoclasts.

It is acknowledged that ROS and RNS, Ca^2+^ ions, ATP, and cytokines are involved in bystander effects (71–73). Extracellular vesicles (EVs) also might play a significant role in these intercellular communications. Exosomes are the smallest (50–150 nm) EVs released by irradiated cells through the fusion of multivesicular endosomal bodies with the plasma membrane (74). EVs are involved in many cellular processes, bystander effects, and activation of the immune system (75, 76). We recently showed that EVs are also are implicated in bystander effects during Auger-based targeted radiotherapy (77).

## Bystander Immunity and Abscopal Effects

Communications of irradiated cells with their microenvironment also include long-distance effects. In the 1950s, the possible role of radiotherapy-induced immune response against cancer cells was suggested. The description of cancer cell death at a distance from the radiation field led to the introduction of the *abscopal* effect concept (78). Briefly, irradiated cells can release damage-associated molecular patterns (DAMPs), including ATP, HMGB1, calreticulin, at the tumor cell surface. In normal conditions, antigen-presenting cells (APC) are present in the blood or in peripheral tissues. According to a precise spatiotemporal pattern, irradiation and the subsequent release of DAMPs can generate a local inflammatory microenvironment that favors the recruitment of immune cells through the secretion of cytokines by macrophages and immature dendritic cells. The subsequent recruitment of APCs (particularly dendritic cells) promotes their phagocytic activity mediated by toll-like receptors, leading to their maturation. Mature APCs express co-stimulating molecules (CD40, CD80, CD86, MHC I and II) of the immune response and chemokine receptors (CCR7) that drive mature APCs to lymph nodes where they prime a specific cytotoxic T lymphocyte-dependent immune response through cross-presentation of tumor-derived antigens to CD8^+^ and CD4^+^ T cells. This corresponds to the so-called adaptive immune response that is specific for dead cell-associated antigens.

Several reports indicate that alpha particle irradiation elicits an immune response. Gorin et al. showed that ^213^Bi irradiated MC-38 tumor cells release DAMPs that activate dendritic cells (79). In mice injected with ^213^Bi-treated MC-38 cells, this induces the adaptive immunity, and an efficient antitumor protection, and therefore alpha particles are an immunogenic cell death inducer, providing an attractive complement to their direct cytolytic effect on tumor cells.

There are also clinical evidences of the immune response involvement in TAT. A preliminary study on 15 men with metastatic prostate cancer without any autoimmune or immune deficiency condition found that ^223^Ra treatment was associated with a lower mean percentage of memory CD8+ T cells that express programmed cell death protein 1 (PD-L1), without any change in CD8+ T cells producing IFN-γ, TNF-α, and IL-13 (80). Another study reported the complete remission of a patient with cutaneous squamous cell carcinoma 76 days after intratumoral treatment with ^224^Ra-loaded seeds. Two other non-treated distant lesions also disappeared, possibly due to an immune-mediated abscopal effect. One year after the treatment, a complete remission of the treated lesion was observed as well as spontaneous regression of the untreated distant lesions [(66); Figure 2]. An ongoing phase Ib study combines the anti-PD-L1 antibody atezolizumab with ^223^Ra (NCT02814669) in metastatic castration-resistant prostate cancer.

Another attractive field actually supporting a more integrated view of radiobiology is the use of TAT to overcome resistance to medications of fungal or bacterial infections (81–84). Although bacteria do not present a nucleus as eukaryotic cancer cells, ^213^Bi-MAb D11 directed against pneumococcal capsular polysaccharide 8 (PPS8) was able to efficiently kill *Streptococcus pneumoniae in vitro* and to reduce bacterial load in C57BL/6 mice (81). Moreover, TAT was also efficient against fungal pathogen such as *Cryptococcus neoformans* by using ^213^Bi-18B7 mAb against capsular glucuronoxylomannan (81). TAT was associated with changes in concentration of the cytokines interleukin (IL)−2, IL-4, IL-10, tumor necrosis factor–α, and interferon-γ, suggesting that the therapeutic effects of TAT may result from changes in the inflammatory response (82). It is noteworthy that, conversely to cancer cells, infected cells are antigenically very different from host cells such that TAT is associated with specificity and low cross-reactivity (83).

It is worth noting also that non-targeted effects could also explain why some studies reported that non-specific control antibodies labeled with alpha-emitters were as efficient as specific ones for treating tumors (85). An hypothesis would be that tumor cells irradiated by the circulating non-specific antibody according to a cross fire mechanism could initiate a bystander and/or systemic response against tumor. This is likely to depend on several parameters such as tumor vascularization and radioactivity tumor uptake.

## Involvement of Cytosolic Double-Stranded DNA in the Systemic Response?

Amongst DAMPs, unrepaired DNA damage in irradiated cells and the presence of cytosolic double-stranded DNA (dsDNA) seem to be critical signals for the establishment of the anti-tumor immunity response. Bioinformatic and meta-analyses highlighted the link between DNA damage repair/response components and mediators of the systemic response as well as the interactions between components of the innate immune response (pattern recognition receptors) and DNA repair proteins (BRCA1, XRCC1, DNA-PK, Ku70/80, and others) (25, 86).

Cytosolic tumor-derived dsDNA is sensed by cyclic GMP-AMP synthase (cGAS) to generate cGAMP required for the activation of stimulator of interferon genes (STING), resulting in the production of interferon-β and induction of several interferon-stimulated genes (87–89). The radiation-induced immunity and toxicities mediated by the cGAS-STING pathway were recently reviewed by Constanzo et al. (90). In addition, the use of high LET particles to trigger the immune response was reported by Durante and Formenti (91).

To date, it is still unclear how cytoplasmic dsDNA is transferred from cancer cells to immune cells, especially to dendritic cells. Transfer via exosomes has been suggested (75, 92). Radiation-induced pro-immunogenic effects in cancer cells are observed in conventional radiotherapy using X-rays with radiation doses from 2 Gy up to 30 Gy or more; however, the optimal radiation regimen to induce a clinically relevant anti-tumor immunity remains to be defined (93, 94). Although radiation-induced cytosolic dsDNA accumulation triggers the cGAS-STING pathway, Vanpouille-Box et al. demonstrated that the absorbed dose delivered to the tumor is critical. Indeed, at doses higher than 12 Gy, cytosolic dsDNA is cleared by three prime repair exonuclease 1 (TREX1), precluding the activation of the cGAS pathway to induce type I interferon, and abolishing the radiotherapy-induced anti-tumor immune response (95, 96). Based on these preclinical results, a phase II clinical trial was started in 2014 in patients with non-small-cell lung carcinoma who progressed after chemotherapy and with at least two measurable disease sites to determine whether radiation (6 Gy × 5 fractions) and immunotherapy (ipilimumab within 24 h of radiotherapy initiation) can stimulate the immune system and stop the growth of tumors that are outside the field of radiation (NCT02221739).

## Conclusion: Consequences of New Paradigms For TAT Radiobiology

The new concepts of TAT radiobiology described in the previous chapters and represented in Figures 1, 2 have several consequences. First, it seems unreasonable to state that only the cell nucleus plays a role in the outcome of irradiated cells and to ignore the other cell compartments. Literature data clearly indicate that all subcellular compartments communicate and that signals produced at the cell membrane or in the cytoplasm can have consequences in the nucleus and vice versa. For example, damaged DNA released in the cytoplasm of irradiated cells can activate immune cells. Irradiated cells also communicate with their neighbors, at short distance (bystander effects) or at longer distance via immune cell activation. These non-targeted effects may have immediate consequences on TAT efficacy (i.e., the probability of cancer cell death increases) and also on healthy tissues. They might also influence the dose-effect relations because cells receiving zero Gy might die.

In bystander effects, cells communicate with neighboring cells via gap junctions or by releasing soluble factors. A plethora of molecules (ROS, nitric oxide, cytokines, ATP, Ca^2+^ etc.) can be involved. EVs also might have a role. As oxidative metabolism is at the center of these signaling pathways, it seems difficult to state that alpha particles only act through direct ionization of DNA, although it might be predominant. We found that bystander effects contributes to 30% of cell killing after irradiation. It is likely that the contribution of non-targeted effects depends on the biological models (tumor, host) but also on physical parameters, including dose and dose rate. Particularly, non-targeted effects might modify the dose-effect relationship. For long time, it was thought that the survival curves of irradiated mammalian cells could be explained by unrepaired DSBs. Therefore, two hits should be required for low LET radiation (which are more likely to produce single-strand breaks), whereas a single hit of alpha particle should be enough to produce this lethal event. However, this is unlikely in term of dose required to produce simultaneously two hits in DNA (97), and also in terms of radiobiology.

## Author Contributions

J-PP and JC wrote the mini-review. All authors contributed to the article and approved the submitted version.

## Conflict of Interest

The authors declare that the research was conducted in the absence of any commercial or financial relationships that could be construed as a potential conflict of interest.

## Publisher's Note

All claims expressed in this article are solely those of the authors and do not necessarily represent those of their affiliated organizations, or those of the publisher, the editors and the reviewers. Any product that may be evaluated in this article, or claim that may be made by its manufacturer, is not guaranteed or endorsed by the publisher.
